# Analysis of the opinions of individuals on the COVID-19 vaccination on social media

**DOI:** 10.1177/20552076231186246

**Published:** 2023-07-10

**Authors:** Akshay Kaushal, Anandadeep Mandal, Diksha Khanna, Animesh Acharjee

**Affiliations:** 1 HSBC Global Research, HSBC Global Banking and Markets, Bangalore, India; 2 Department of Finance, 150183 Birmingham Business School, 1724 University of Birmingham, Birmingham, UK; 3 Institute of Cancer and Genomic Sciences, 1724 University of Birmingham, Birmingham, UK; 4 Software Developer, BPD Zenith Limited, London, UK

**Keywords:** COVID-19, vaccinations, social media, sentiment analysis, machine learning

## Abstract

The COVID-19 pandemic continues to threaten public health globally. To develop effective interventions and campaigns to raise vaccination rates, policy makers need to understand people's attitudes towards vaccination. We examine the perspectives of people in India, the United States, Canada, and the United Kingdom on the administration of different COVID-19 vaccines. We analyse how public opinion and emotional tendencies regarding the COVID-19 vaccines relate to popular issues on social media. We employ machine learning algorithms to forecast thoughts based on the social media posts. The prevailing emotional tendency indicates that individuals have faith in immunisation. However, there is a likelihood that significant statements or events on a national, international, or political scale influence public perception of vaccinations. We show how public health officials can track public attitudes and opinions towards vaccine-related information in a geo-aware manner, respond to the sceptics, and increase the level of vaccine trust in a particular region or community.

## Introduction

The COVID-19 virus has had a substantial impact on our day-to-day lives,^1^ as well as our bodily and mental health, the state of the environment, and our ability to provide for ourselves financially.^2,3^ Numerous people have lost their lives all around the world because of the disease. The effects that it will have on both the economy and society will be unfathomable.^4^ As a consequence of these devastating losses and the disheartening information on COVID-19's effects, the mental health of the people has taken a significant hit.^1,5,6^ As a direct consequence of this, the general mood is one of discontentment, sadness, and disappointment.^7,8^ Therefore, it was only normal for people to be sceptical of the COVID-19 immunisations that were being provided to them.^9^ People questioned the effectiveness of vaccinations as well as whether appropriate safety measures were taken during the manufacture of vaccines.^10–12^ The public is hesitant to participate due to a variety of causes, including uncertainty, problems in registering or scheduling, fear of potentially unfavourable outcomes, and other considerations.^13,14^

**Table 1.** Table providing details on the 40 COVID-19-related subreddits/communities selected from Reddit for data collection.

S.No.	Subreddit/Community	Country	No. of Posts
1	coronavirusindianews	India	812
2	covidIndia	India	380
3	IndiaCovid19	India	146
4	covid19inIndia	India	112
5	COVID_19India	India	88
6	IndiaVsCorona	India	72
7	Coronavirus_India	India	54
8	indiacorona	India	28
9	coronavirus_inIndia	India	6
10	ukantilockdown	UK	2000
11	CoronaUK	UK	2000
12	CoronavirusUK	UK	1980
13	CovidLongHaulersUK	UK	92
14	NoNewNormalUK	UK	36
15	UKCovid	UK	20
16	CovidRebellionUK	UK	12
17	UKCovidShielding	UK	12
18	COVID_CANADA	Canada	2000
19	Coronavirus_BC	Canada	2000
20	CanadaCoronavirus	Canada	1996
21	CoronaVirusMontreal	Canada	1994
22	CoronavirusCanada	Canada	1988
23	CoronavirusOntario	Canada	792
24	CovidCanada	Canada	644
25	CanadaCOVID	Canada	368
26	CanadaCOVID19	Canada	82
27	CoronavirusUSCOVID19	USA	2000
28	CoronavirusFlorida	USA	2000
29	coronavirusNYC	USA	1998
30	CoronavirusCalifornia	USA	1998
31	CoronavirusUS	USA	1976
32	CoronavirusNewYork	USA	1932
33	CoronavirusLA	USA	1922
34	CoronavirusTX	USA	1884
35	CoronaNC	USA	1774
36	CoronavirusSanDiego	USA	1528
37	Covid19_USA	USA	1498
38	CoronaUSA	USA	1054
39	AmericanPandemics	USA	1000
40	PoliticalCovid19	USA	592

The patients have a range of feelings in relation to their medical treatment. Nearly every interaction a patient has with a healthcare provider, whether they are a doctor or a hospital employee, will elicit sensation. As a result, sentiment analysis has a tremendous amount of use in the healthcare sector.^6^ Examining the perspectives of the patients may assist the medical staff in breaking down the barriers that prevent effective communication between hospitals and their patients. Because of this, they can greatly improve the results of the organisation as well as the satisfaction of their patients.

The continual discussion over vaccination developments, accessibility, effectiveness, and side effects dominates the daily headlines in the media and the domains of Twitter.^15^ Despite this, internet users are only allowed limited access to the website.^16^ Consequently, the purpose of this research is to make use of the data provided by social media platforms such as Twitter and Reddit,^17^ (please see Table 1) in order to get a more comprehensive comprehension of the current state of the worldwide pandemic.^12^ When it comes to COVID-19 vaccinations, it would be challenging for humans to comprehend and get a handle on any information. However, by using natural language processing (NLP)^18^ methods like textual information collection, emotion analysis, and visualisations with word clouds, we can examine a topic that is both very complicated and vast.^19^

Many studies have made use of social media, surveys, and machine learning to analyse the public opinions and perspectives across various countries in the past. For instance, a study conducted in India in 2021 showed that 78.5% of the tweets had either positive or neutral sentiment towards the side effects of various COVID-19 vaccines.^20^ On the similar lines, another study conducted in India during 2021 showed that only 35% of tweets had a positive tone towards COVID-19 vaccines.^21^ Another study conducted surveys across many countries and found a considerably high willingness to take COVID-19 vaccines across individuals in 10 low and middle income countries in Asia (80.3%) compared with the United States (64.6%) and Russia (30.4%).^22^ However, as per one study conducted across 114 countries/territories in 2022, Canada had one of the highest vaccine acceptance rates (91%) compared with India (79%), the United States (66%), and the United Kingdom (81%).^23^ While the hesitancy related to vaccine acceptance still remains,^24^ some of the major concerns have been observed around the side effects and effectiveness of vaccines,^25,26^ distrust in scientific community,^27^ and misinformation on the internet.^28,29^

In this study, we expand the findings of past research on how people respond to and comprehend COVID-19 vaccinations via the social media. During the social isolation and lockdowns that were caused by COVID-19, many users had little option but to vent their views on social media sites such as Twitter and Reddit.^30,31^ Further, social media provides an opportunity for private communication between companies and their end users. Because there is so much data available on social media, it may be difficult for marketers to identify mentions that have the potential to have an effect on their company.^32^ Hence, with this paper, we mainly aimed to determine the following: (1) Is there a difference in public sentiments towards various COVID-19 measures taken across four countries viz. Canada, India, the United States, and the United Kingdom? (2) Is there a difference in public sentiments towards various technology of vaccine (vaccine option) across four countries? (3) What is the overall trend in perceptions on COVID-19 vaccines in these four countries under consideration?

**Figure 1.**
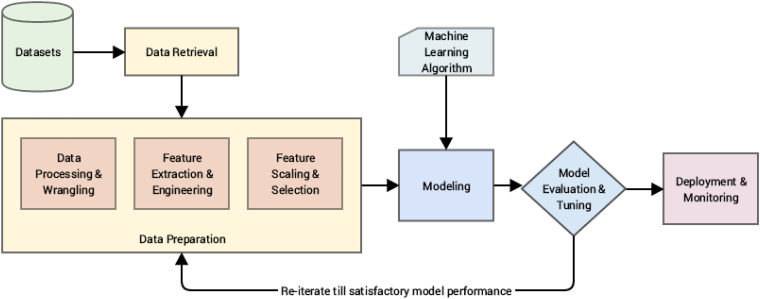
Infographic abstract representing the proposed machine learning pipeline with respect to the described cross industry standard process for data mining (CRISP-DM)^38^ framework. The figure above shows various steps involved in the machine learning pipeline proposed in this study beginning with data selection and retrieval followed by data preparation, modelling, and evaluation before final deployment and consistent monitoring. Application of several machine learning algorithms was assessed at the modelling and evaluation stage, and the process was re-iterated using various models until a satisfactory result was obtained.

This study evaluates the relationship between public opinion and emotional dispositions towards different COVID-19 vaccinations and trending topics on Twitter and Reddit in India, the United States, Canada, and the United Kingdom. In addition, machine learning algorithms such as Naive Bayes,^33^ support vector machine (SVM),^34^ K-nearest neighbour (KNN),^35^ and logical regression^36^ were trained to predict these ideas based on social media posts (Figure 1). Utilizing three Kaggle-collected data sets from Twitter and one data set collected from Reddit, the research analyses sentiment and mood patterns in society at various times and locations. Using emotion analysis (with keyword cloud mapping^37^), and modelling, we were able to discover important perceptual occurrences and patterns.

The study acknowledged not only the common sentiment classifications of positive, negative, and neutral, but also the anticipated challenges of utilizing machine learning. Thus, we used artificial intelligence to perform text categorisation to interpret the people's perspectives on vaccination rather than relying on the traditional sentiment classifications. Given that the objective was to do a multi-class classification, we trained our models to divide the sentiments expressed in a post into two buckets (positive and negative) to perform modelling using different classifiers. It was revealed that a support vector classifier had the best accuracy, which was 87% when it came to the classification of two different classes.

With this study, first we show that since people tend to trust public figures because they are elected officials with the authority to change healthcare systems and have more knowledge regarding vaccinations,^39,40^ politicians have a significant influence on popular anti-vaccination beliefs.^41^ Hence, opponents of vaccination who make derogatory comments may convince some people, especially those with an open mind.^42^ Second, our data shows that anti-vaccination conspiracy beliefs decreased general sentiment. Mainly because social media platforms with large user bases have ‘disrupted’ the established means of delivering vaccine information,^43^ making it simpler for anti-vaccination advocates to spread incorrect information to a vulnerable audience. Finally, sensation and emotion ratings revealed country-specific trends. It is easy to find people with negative sentimental and emotional assessments who require additional study to understand the public's anxieties about COVID-19 injections.

The rest of the work is organised as follows: methodology used in this study and the data are discussed in the *Material and methods* section. The output of the data pre-processing, sentiment analysis, and the study of the data set using machine learning classifiers are included in the *Results* section. This is followed by a comprehensive discussion of major findings, implications of the study, limitations, and direction to the future studies in the *Discussions and conclusion* section.

## Material and methods

### Data collection

We use four distinct data sets to have a wider perspective on different perceptions of people. Three of the data sets from Twitter have been contributed by the members of the Kaggle community while we have additionally collected one data set from Reddit separately. The details of the data sets are given below:

*COVID-19 all vaccines tweets.* The most recent tweets on the COVID-19 vaccines Pfizer/BioNTech, Sinopharm, Sinovac, Moderna, Oxford/AstraZeneca, Covaxin, and Sputnik V were collected and compiled by Preda (2021).^44^ The information is gathered with the help of the Tweepy Python package, which connects to the Twitter application programming interface (API). Each immunisation required its own unique set of search terms, which Preda (2021) performed (most frequently used in Twitter to refer to the respective vaccines). The data has 228,207 observations (tweet chats of each participant) and 16 variables.

*COVID vaccine tweets.* Kash (2022)^45^ describes the data collection procedures for this data. He first creates a twitter account, creates a twitter app using the link: https://apps.twitter.com/app/new, and set up the authentication and connection with Python environment. Tweets are extracted with the #COVIDVACCINE hashtag. The data has 397,598 observations (tweet chats of each participant) and 16 variables.

*COVID-19 vaccine tweets.* Yadav (2020)^46^ uses similar data mining as Kash (2022) and Preda (2021).

*COVID-19 posts from Reddit.* We use Pushshift Reddit API^47^ to collect approximately 42,000 posts made by various users in 40 COVID-19 related communities across four countries. We searched for words such as ‘Covid’ and ‘Corona’ on Reddit, and selected the top 40 subreddits/communities in total based on relevance from all four countries: India (9), the United Kingdom (8), Canada (9), and the United States (14). Finally, we used the following search terms ‘vaccine’, ‘dose’, ‘jab’, ‘booster’, ‘moderna’, ‘pfizer’, ‘covaxin’, ‘covishield’, ‘sputnik’, ‘oxford’, ‘janssen’, and ‘novavax’ to filter out the relevant posts.

Each of these data sets included tens of thousands of posts pertaining to the COVID-19 vaccination. The posts were organised into categories according to the locations from which they were tagged. At first, it was seen that the tweets originated from a variety of nations; while some only identified a city or region, others simply referred to the name of the country itself. The location of origin was altered so that the selected tweets would only carry the name of the country of origin, except for the tweets coming from India, which maintained the region as the origin location for some reason. Also, one limitation of the data set remains in terms of its representativity; notably, young people who are active users of the internet and mobile apps make up the vast majority of those who participate in social media. Hence, these users do not provide an accurate representation of the views and attitudes held by the larger community, which is comprised of people from a variety of racial, ethnic, and socioeconomic backgrounds.^48,49^

## Cross-industry standard process for data mining method

The cross-industry standard process for data mining (CRISP-DM) method^50^ in the form of a hierarchical process model was used on the Twitter and Reddit chats obtained. This model consists of sets of tasks that are each described at one of four levels of abstraction, from more general to more specific: phase, generic task, specialised task, and process instance.^38^ At its most fundamental level, the data mining process may be broken down into several steps, each of which is made up of a specific collection of activities. The components of the methodology used in the study are further described in the subsequent sub-sections.

### Data pre-processing

The pre-processing of the data involved the following steps: (1) *Loading the data;* this involved using the python package ‘pandas’, with the low memory option as false, since the data sets were huge; (2) *Setting the correct data type* for each of the important variables. This was done on the variable ‘text’, from float to string; (3) *Cleaning the posts;* this involved removing links, web addresses, email IDs, etc. Further, uppercase words and letters were set to lowercase. Finally, in this step, words were tokenised. For languages other than English, this refers to dividing a big text into fewer lines, words, or even single words.^51^ As can be seen below, applications may utilise the different tokenisation features provided by the NLTK module. For example, initially, a text read: ‘The agency also released new information for h … ’ and after cleaning remained as: ‘agency also released new information health ca … ’; (4) *Lemmatisation;* this is the process of merging different word spellings.^52^ The users may search for any variation of a root word using lemmatisation and get relevant results; (4) *Location specification;* since the posts are from different location, only the location with the following labels were selected: India, UK, USA, and Canada.

### Feature extraction

**Figure 2.**
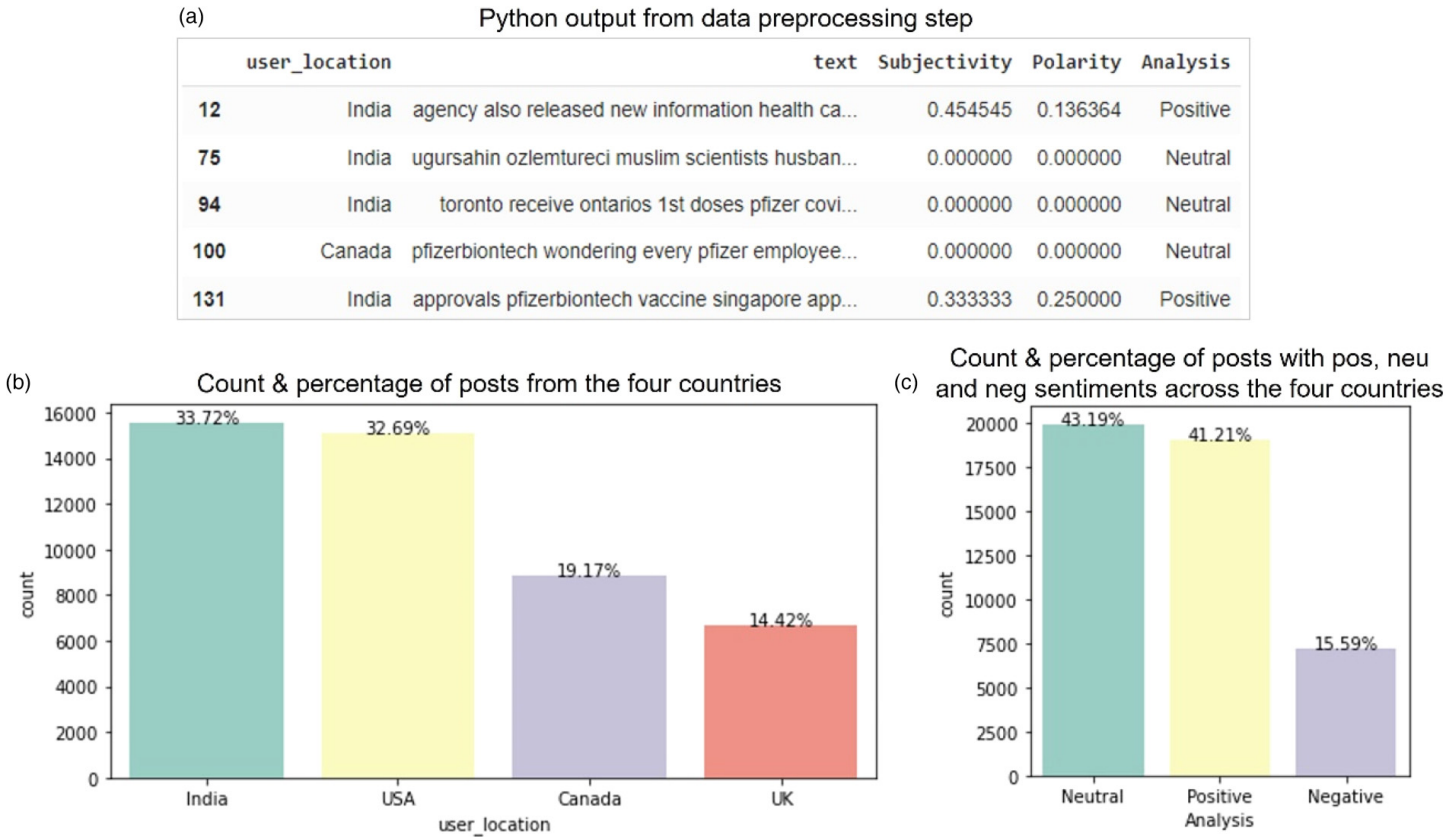
Data pre-processing and preliminary exploration. The figure on top shows the sample output from the data pre-processing step: (a) The distribution of posts in India, the United States, Canada, and the United Kingdom in the merged data has been provided in (b) whereas the sentiments distribution within the four merged and pre-processed data sets is shown in (c).

To employ machine learning algorithms to classify (predict) sentiments, we only use two labels (positive taking a value of 0, and negative taking a value of 1). The data has a total of 13,668 observations and two columns. To perform numerical analysis on the text data using machine learning algorithms, our documents first needed to be converted into vector representations. The initial stage in any language-aware analysis must always begin with feature extraction, and in our case, the study used vectorisation using the TfidfVectorizer^53^ with n-gram range of (1,3), i.e. a trigram. Vectorising the texts gave 145,789 features. Also, it is seen that the distribution of both labels is significantly different from each other (Figure 2(c)). And so, there is no need to scale the labels and splitting and sampling are possible.

### Sentiment analysis

Sentiment analysis has been employed for gaining an understanding of the author's intended tone in a piece of writing. Following the cleaning up and pre-processing of the data, the polarity value of each post was determined. Polarity is a kind of floating-point number in the range,(–1,1), where 1 represents a positive statement and −1 a negative one.^54^ By analysing each post using the polarity measure that was developed, we were able to determine if it included positive, negative, or neutral emotion. Therefore, the posts whose polarity values were more than zero were categorised as positive, the posts whose polarity values were equal to zero were labelled as zero, and the posts whose polarity values were less than zero were categorised as negative. Even though the data sets that were utilised included information on a post's user, location, date, text, and hashtags, for the sake of this study, only the text and label elements were kept. We also calculate the subjectivity scores, which often relate to opinion, emotion, or judgment, whereas the objective ones pertain to observable facts.^55^

### Machine learning algorithms

This study's objective is to identify the most common feeling conveyed by the users of the social media platform Twitter and Reddit. Several different categorisation methods were used, which allowed the sentiment of a post about COVID-19 immunisations to be deduced. The identification of different classes or groups is the primary goal of the categorisation process. Polarity was an important consideration in the classification strategies that we used because each classifier functions in a unique way.

$$Polarity=\frac{\frac{P(Positive\;Sentiments)}{P(Total\;Sentiments)}}{\frac{P(Negative\;Sentiments)}{P(Total\;Sentiments)}}$$

It was then assigned three labels, ‘Positive’ for when polarity is more than 0, ‘Negative’ for when polarity is less than 0, and ‘Neutral’ for when polarity is 0. For algorithms, this study uses only two labels to classify perceptions: Positive and Negative.

Machine learning algorithms used in this study are KNN, logistic regression, Naive Bayes, and SVM. The models were trained using a 70%–30% ratio, creating the train and the test sets. These were compared from each other to find the most accurate predictor. In the case of KNN, we employ *five-fold cross-validation*^56^ and *RandomizedSearchCV*^57^ to determine the best estimate for the number of neighbours in a list of values ranging from 2 to 25. We found that the model achieved highest accuracy when the number of neighbours was set at 7. Considering logistic regression, we employed L2 regularisation^58^ and penalised the model by adding the squared magnitude of coefficients to the loss function. Further, we use Multinomial Naïve Bayes classifier mainly because it is relatively better suitable for text-based classification tasks and usually works well with fractional feature counts such as Tfidfvectorizer.^59^ For SVM, we employ *five-fold cross-validation* to enable probability estimates and set the value of the regularisation parameter *C* as *100* and *kernel* as *linear* with the value of *gamma* as *0.01* and *class weight* as *balanced.*^60^

Additionally, in order to explore the underlying data efficiently in more depth, we employ Sentence Transformers,^61^ Facebook AI Similarity Search (FAISS),^62^ and text analysis techniques. We extract the features of the text using Sentence Transformers to build a FIASS index of the whole data set. It allowed us to collect the samples of posts that are most closely related to any search-term under consideration based on the Euclidean distance, and explore it further in more details.

### Ethical considerations

Twitter and Reddit approved for the study and provided access to their API, which was used to collect posts. Because all the gathered posts are already part of the public domain and can be seen by anybody, there was no need for an ethical assessment to be conducted. However, this research handled the data with the utmost care, to the highest ethical standards throughout the process. No posts (and user information) were read or used in any manner for this research.

Although a significant number of posts were retrieved, all personally identifiable information as well as the substance of each post was scrubbed once the average daily sentiment was computed.

### Data and scripts availability

The raw data used in this study is available publicly on Kaggle^44–46^ and GitHub.^63^ More details related to it have been provided in the *Data collection* sub-section. The coding scripts used to produce the results are also made available separately in a public repository^63^ - https://github.com/akshaydnicator/covid19perceptions.

## Results

### Data exploration

After the pre-processing step, the first five rows of the data looked as shown above (Figure 2(a)). The distribution of the user location and their countries was also studied. As shown above (Figure 2(b)), India (about 34% of the filtered posts, which is more than 15,500 posts), the Unites States (about 33% of the filtered posts, which is more than 15,000 posts), the United Kingdom (about 14.4% of the filtered posts, which is about 6600 posts), and Canada (about 19% of the filtered posts, which is more than 8800 posts) were the only four nations that were finally taken into consideration.

### Sentiment analysis

The percentage of posts in each sample that were negatively categorised was, on average, 15.6% of the total number of posts, 43.2% on neutral posts, and 41.2% on the positive posts. From the total of 46,140 posts in the final data set after the pre-processing process, the figure above (Figure 2(c)) depicts the total number of posts that were classified as either positive, neutral, or negative sentiments. This shows that most people in the study were undecided on the COVID-19 vaccine.

**Figure 3.**
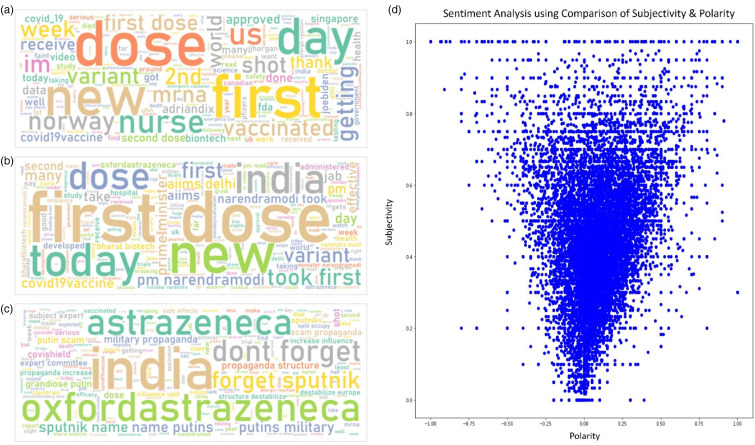
Visualising the output of the sentiment analysis using word clouds and scatter plot. We develop word clouds to visualise the top keywords in all the posts combined (a), and for the posts with positive sentiments (b) and negative sentiments (c) separately. Further, we visualise the polarity and subjectivity scores of all the collected posts using scatter plot (d).

The word cloud shown above (Figure 3(a)) is depicting the topmost often occurring terms in the posts. The word cloud reveals that the most appearing words show relatively more positive sentiment than negative. From the posts with positive sentiments shown below (Figure 3(b)), the first impression of positivity in the word cloud is ‘took’, ‘jab’, ‘many’, ‘health’, ‘good’, ‘story’, ‘true’, and ‘effective’. Furthermore, words like ‘Propaganda’, ‘scam’, ‘forget’, ‘allergic reaction’, and ‘fake’ (Figure 3(c)) show negative perceptions about the COVID-19 vaccination.

Subjectivity and polarity were used to further compare perceptions. To compare these metrics and determine the general trend of perceptions, we visualise the two using a scatter plot (Figure 3(d)). From the figure, we can see that points are concentrated around subjectivity 0.4 and polarity 0.2. Interpreting this, we say that majority of the observations are positive and public opinion than factual information.

**Figure 4.**
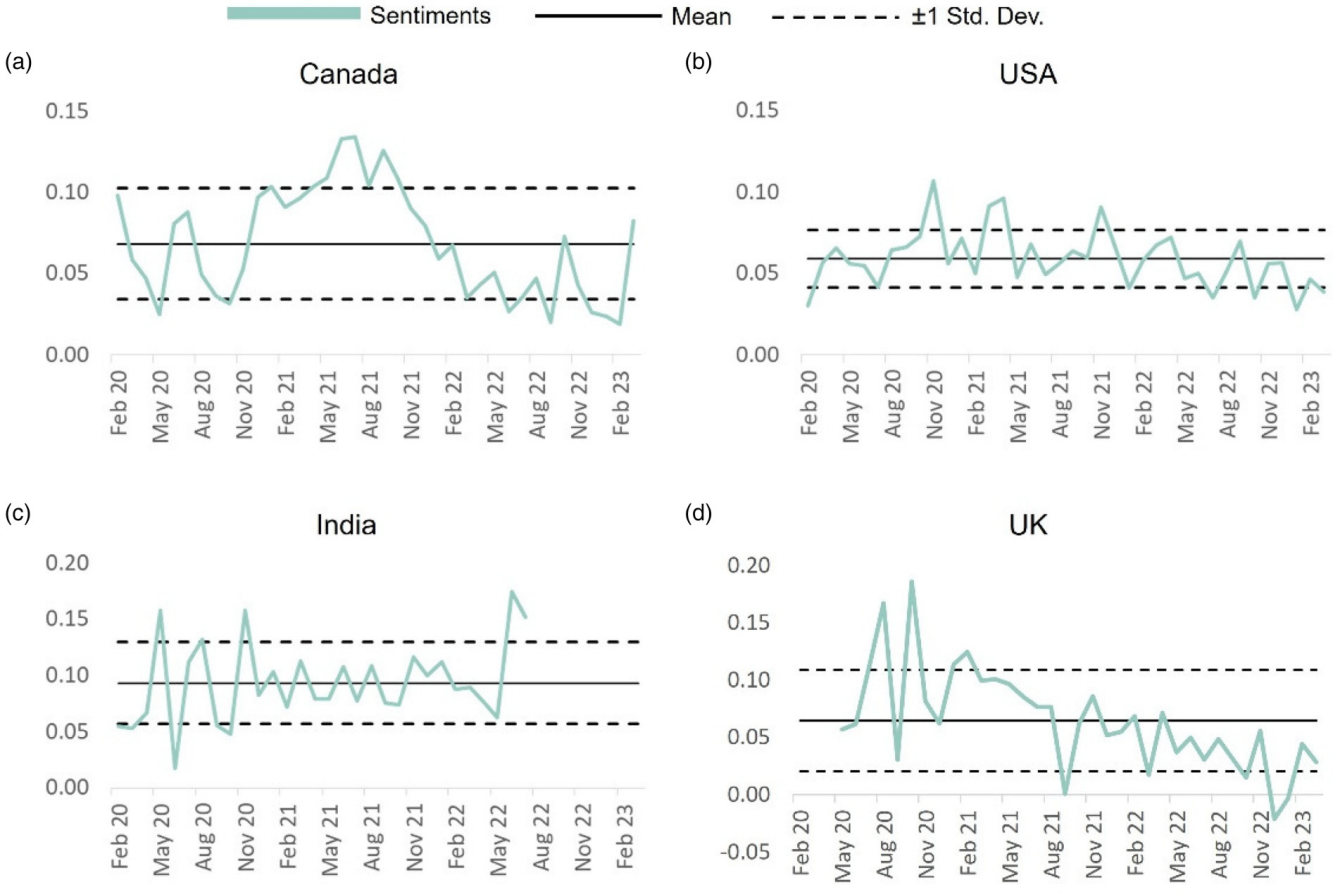
COVID-19 vaccination sentiments evolution across the four countries. The line charts depict the evolution of the public sentiments towards COVID-19 vaccination from Feb 2020 to Feb 2023 across Canada (a), the United States (b), India (c), and the United Kingdom (d).

Overall, we observe that India had the most positive outlook towards COVID-19 vaccines over time followed by Canada, the United Kingdom, and the United States in that order (Figure 4). Canada experienced highest emotional variance among the four countries with the positive sentiments following a somewhat Transverse wave pattern reaching its peak in June 2021 and hitting its lowest in January 2023. The United States and the United Kingdom however followed a similar trend with sentiments rising significantly in the first year (2020) and following a general downtrend thereafter. Considering that the cultural differences and social norms have a considerable impact on an individual's behaviour and opinions,^64^ we relate our findings to the classic individualism vs. collectivism debate.^65^ On the one hand, we have countries such as Canada, the United Kingdom, and the United States that are mostly argued to have loose cultures and higher individualism as a result.^66^ On the other hand, we have India that has relatively strict cultures and higher collectivism as a consequence. Since in the countries with strict cultures people stay in tight communities,^67^ we observe that even though higher collectivism is associated with higher risk of outbreak,^68^ it also makes people more compliant and provides them with an opportunity to encourage one another and boost their sentiments as a result. On the contrary, in the countries with loose cultures, people prioritise individual freedom and privacy.^69^ As a consequence, even though higher individualism is associated with lower risk of outbreak, it has a negative association with compliance to guidelines such as the social distance practice,^70^ as people tend to be more individualistic in nature and lack peer pressure. Hence, we clearly show that while both the loose and strict cultures have pros and cons of their own, there is no clear winner when compared in the context of COVID-19.

### Analysing the positive sentiments on the COVID-19 vaccines

Compared with posts from other nations, those from India were much more upbeat and encouraging. In India, where there were relatively few cases during the first few months of 2021, the general population very certainly held the impression that the epidemic had passed its peak. During the month of May, the number of daily cases skyrocketed to an unsustainable high before gradually declining to an average of around 40,000 cases per day. As the initial elation faded and profound sorrow set in, the percentage of those who were experiencing good sensations fell.

Considering the United States, it had varying reactions to the COVID-19 epidemic, mainly because of decentralised limitations that were implemented by each state. During the epidemic, there were demonstrations and marches held in opposition to the stringent lockdown regulations and the obligation to wear a mask.^71^ On the other hand, the federal government was also actively urging public distance and other safety measures.^72^ These conflicting feelings did not significantly alter the proportions of positive sentiment, but a sizeable number of those who started out with a neutral attitude later shifted to having a negative attitude.

It is evident that various stages of the epidemic have distinct effects on the general public's mindset. It was anticipated that following the launch of vaccines, most posts would be positive towards them. This was because the World Health Organization (WHO),^73^ the Centers for Disease Control and Prevention (CDC),^74^ and other international public health organisations including Indian Council of Medical Research (ICMR)^75^ were promoting the vaccines. In India, immunisation efforts were not earnestly carried out until January 2021, when it received its first shipment of vaccines.^76^ By April of 2021, it was available to a sizeable section of the population over the age of 45. After that except for the posts coming from India, the proportion of posts that were supportive of vaccines went down in other countries with time. When compared with India that saw a significant rise in positive sentiments towards COVID-19 vaccination during June–July 2022, a sharp drop was observed across Canada, the United States, and the United Kingdom, with only Canada recovering to some extent by February 2023 (Figure 4).

The advent of the second wave, which swept across the globe beginning in the middle of April 2021 and lasted for several weeks, provides an explanation for the fall in vaccination support in India.^77^ The public health system buckled under the weight of an incredible amount of strain. The immunisation push was hindered since there was a scarcity of doses, a shortage of workers, and a stressed setting.^78,79^ The percentage of people who have a neutral or positive outlook climbed from 38.5% to 61.5%, while the percentage of people who have a negative outlook declined from 7.5% to 11%.

Throughout vaccine deployment in the United Kingdom, issues were identified. It is possible that this contributed to a short-term shift in how people feel about being vaccinated. Bear in mind that at the same time, there was a distinct decline in negative attitude and, similarly to India, there was a visible rise in neutral attitude.

The study by the Institute of Policy Research at Northwestern University^80^ found that the Republican governors were opposing vaccination rules in a number of states inside the United States as they were becoming increasingly suspicious about vaccinations. This potentially caused a sharp decline in positive sentiments in the United States post November 2021.

When compared with the other four nations, Canada had a disproportionately high number of pessimistic views in the month of July 2021, and it grew progressively since then. This was triggered by the increase in COVID-19 infection during the second wave. In contrast to the other nations, the United Kingdom witnessed a rise in the number of people expressing good feelings while simultaneously seeing a drop in the number of people expressing negative feelings. This was potentially due to an efficient administration and roll out of the vaccination programme and patients witnessing less severe symptoms and effects when infected by COVID-19 virus.

### Analysing the negative sentiments on the COVID-19 vaccines

Numerous persons ‘warned’ that vaccines generally ‘took 10 years’ to develop and that a vaccine developed during the first few periods was dangerous.^81^ Some people make the decision not to be vaccinated because they are concerned that an ‘untested’ and ‘experimental’ vaccine would be made accessible to the public in the form of ‘laboratory rats’ and ‘guinea pigs’. The users rated the severity of instant negative effects higher than that of the long-term negative effects. Some examples of immediate negative consequences include ‘skin peeling’, ‘horror deaths’, ‘facial paralysis’, and ‘blood clots’. The fact that several commenters brought up the safety concern that delaying the second dose was dangerous since it was ‘off-label’ and ‘contrary to scientific advice’ triggered such public opinions and comments. Those who had been vaccinated posted about their unpleasant effects, such as ‘sore arm’, ‘symptoms like the flu’, and ‘headache’, and a very small number of them encouraged others to opt out of being vaccinated.

Most posts that triggered distrust referred to the pharmaceutical industry and/or other goals of the government. There were a few posts that included conspiracy theories, such as the assumption that the government is using mass vaccination to weaken the immune systems of ‘sheep’ and that pharmaceutical companies are purposefully creating or exaggerating the COVID-19 outbreak to increase sales of vaccines. Other posts stated that the government is using mass vaccination to weaken the immune systems of ‘humans’, and others expressed concerns about the government's capacity to carry out the vaccination-rollout, citing ‘test and trace’ and ‘care homes’ as examples of previous mistakes made during the first outbreak to justify their lack of faith in the government's ability to do its job effectively. Several individuals voiced their opinion that the emergence of new ‘mutant strains’ or ‘new variants’ is a ‘government myth’ that is intended to conceal the reality that vaccines ‘never truly worked at all’. This mistrust was bolstered by the dissemination of a news article that asserted ‘big pharma’ companies were ‘protected’ from ‘being sued’ or taking any ‘legal culpability’ for adverse effects caused by vaccinations.

It was claimed, among other things, that vaccinations were ‘less effective than promised’, that they were ‘ineffective against mutations’, and that postponing the administration of the second dose would result in a reduction in the level of protection over time. However, from such statements, it is difficult to say whether people were motivated to reject vaccination, arising from their lack of knowledge about the effectiveness of the procedure. The scepticism that immunisations would not fix the disease as promised contributed significantly to the negative tone that was shown in the posts addressing the topic. It is essential to keep in mind that this is not the standard practice, even though most blogs that brought attention to the fact that one may ‘still develop COVID’ despite being vaccinated used this information to argue against the controversial vaccination passports rather than as a reason not to get vaccinated. However, a small percentage of users questioned the benefit of getting a vaccination that was deemed ‘ineffective’ since it did not prevent the spread of COVID. This indicates that continuing disagreement about the vaccine's lack of efficacy deterred individuals from acquiring it.

We show that the difficulties of obtaining immunisations garnered a significant amount of attention. However, as seen by several posts, a significant number of people who support vaccination are incensed by ‘queue jumpers’, particularly those who are not considered ‘vulnerable’, as opposed to the true problems with access to vaccinations. The posts that were coded for accessibility often revealed the users’ feelings of dissatisfaction with the ‘time-consuming’ or ‘complicated’ nature of appointment scheduling. When attempting to arrange an appointment using the NHS website, some users voiced their dissatisfaction that the process was ‘harder than purchasing Glastonbury tickets’. According to a few posts, the customers disregarded NHS text message appointment reminder notifications because they believed they were ‘scams’ or ‘fake SMS’ related to vaccinations. Therefore, the customers missed their scheduled appointments.

A great number of posts highlighted the need of being vaccinated. In response to articles in the news regarding vaccines, the users often posted responses that included the terms ‘what's the point?’ or ‘no need’. Some others claimed that the widespread use of vaccinations was an ‘overreaction’, and that those who are the most at risk should be the only ones receiving protection. The apathy of some users was made clear by their comments that they were ‘done with COVID’ and that all they want is for things to go back to normal.

In fake posts, the safety, reliability, and efficacy were given a great deal of emphasis. We witness that a significant number of posts had incorrect information. This might have been because of user ignorance, or it could have been the result of posts by the so-called ‘anti-vaxxers’, who purposefully propagate misinformation to discourage vaccination. The people who did not believe that the coronavirus pandemic was real made numerous posts in which they referenced anecdotal ‘proof’ of how dangerous the vaccinations were. Even while the bulk of posts containing wrong information seemed to originate from ‘anti-vaxxers’ or those who are sceptical about vaccination, only a very small fraction of the users provided fake information to promote vaccination. For example, several posts spread misleading information about the relative risk of developing blood clots after getting the AstraZeneca vaccination compared with the risk of developing blood clots after taking the pill. The fact that a significant number of the posts in our sample included incorrect information highlights the possibility of difficulties, even though social media platforms are an essential medium for the dissemination of health information. Public health is put at jeopardy when online groups are encouraged that disregard the advice of professionals and base their healthcare decisions on incorrect information found on the internet.^82^

### Analysing the sentiments towards various attributes of COVID-19 vaccination

**Figure 5.**
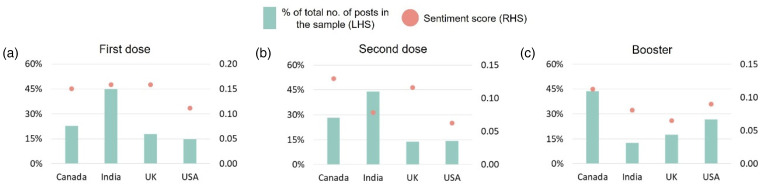
Comparing the sentiments towards the different COVID-19 doses administered across countries. On the left hand side of the subplots in the *y*-axis, we show the percent of total number of posts across countries in the respective samples collected from the FAISS index using similarity search. And on the right-hand side, we show how the sentiment scores vary across countries by the type of dose administered.

In order to compare different attributes of COVID-19 vaccination, we employ Sentence Transformers and FAISS index to collect the relevant samples from the merged data using similarity search. First, we compare the four countries based on the sentiments towards the different types of vaccine doses administered, i.e. first dose, second dose, and booster (Figure 5). Overall, we observe a decrease in positive sentiments across all of the four countries as the vaccination process progressed alongside the first dose, second dose, and booster. The posts from Canada largely had relatively positive outlook throughout the vaccination process as compared with other countries, and the proportion of posts from Canada discussing various vaccine doses also increased with each progression thereby showing that more and more users from Canada were expressing their opinions on vaccine doses on social media as vaccination progressed. However, in the case of the United Kingdom, the proportion of posts remained somewhat stable, whereas in the case of India and the United States, the proportion of posts remained stable through the first and second doses but the representativity significantly reduced from India and increased from the United States during the booster dose administration mainly because the booster dose roll out was delayed in India by 3 months.^83,84^

**Figure 6.**
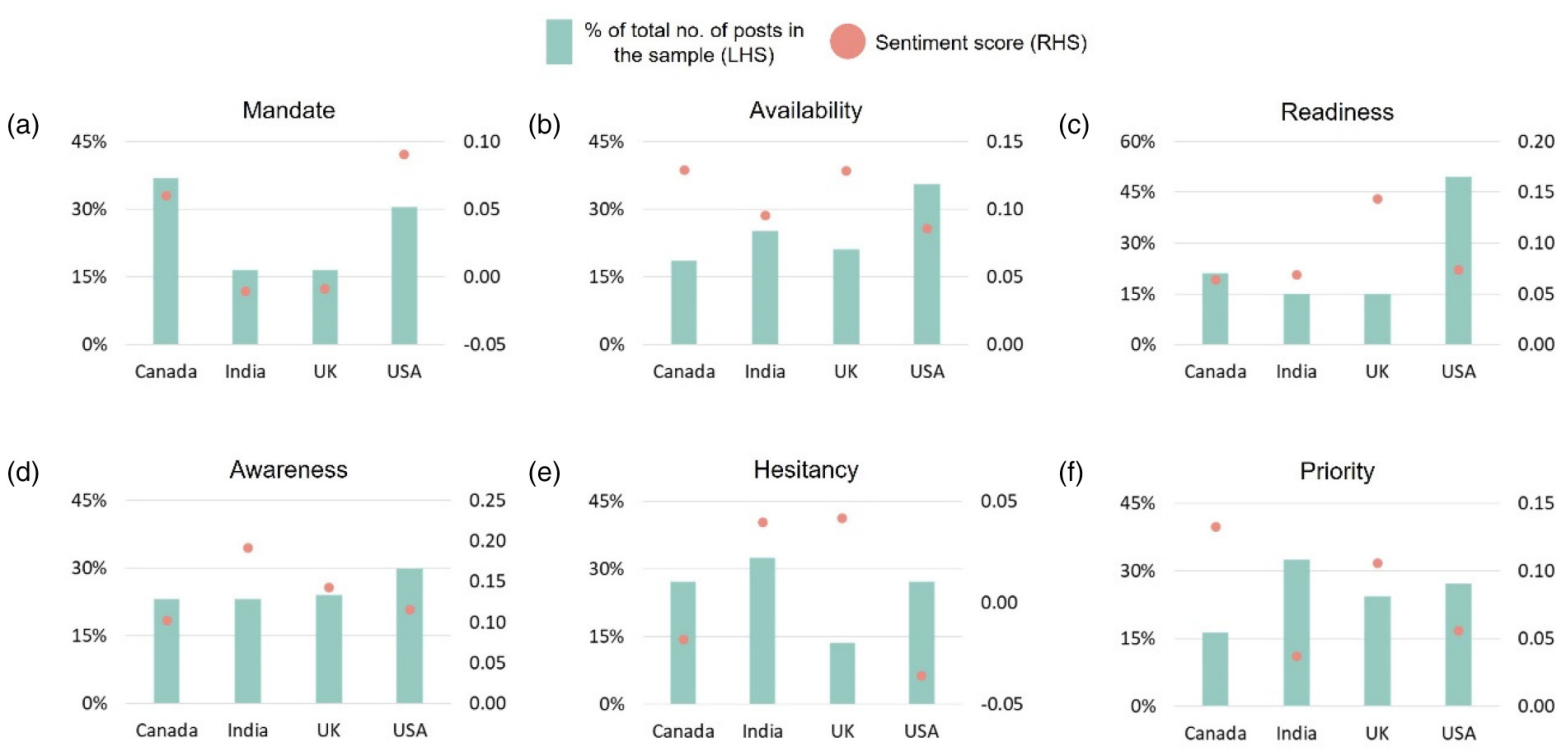
Comparing the sentiments towards the different COVID-19 vaccination-related policy decisions, strategies, and attributes across countries. On the left-hand side of the subplots in the *y*-axis, we show the percent of the total number of posts across countries in the respective samples collected from FAISS index using similarity search. And on the right-hand side, we show how the sentiment scores vary across countries on the basis of the different COVID-19 vaccination-related attributes and policy decisions implemented.

We further compare some of the key COVID-19 vaccination-related attributes and policy decisions that are common across the four countries (Figure 6). One such policy decision was to make the vaccination doses *mandatory* for employment and travel (Figure 6(a)). In this case, we observe that not only Canada and the United States dominate in terms of higher proportion of posts discussing the vaccination mandate but they both had somewhat positive outlook towards it as opposed to India and the United Kingdom that had lower proportion of posts and negative outlook towards the mandate. Another policy decision around COVID-19 vaccination was to enable infrastructure to make sure the *availability* of the vaccine for the broader population (Figure 6(b)). In this case, the United States had the highest proportion of posts discussing the vaccine availability while having the lowest sentiment score at the same time, thereby signifying that the users from the United States were most concerned with the availability of the vaccine as compared with the other three countries. Another important COVID-19 vaccination policy decision relates to the *readiness* of the vaccines and infrastructure (Figure 6(c)). In this case, while Canada and India had both relatively lower proportion of posts and sentiment scores towards COVID-19 vaccine readiness, the users from the United States showed the most concern with the highest proportion of posts and relatively lower sentiment score, whereas the users from the United Kingdom showed least concern with the lowest proportion of posts and highest relative sentiment score.

Further, we compare the four countries based on the policy decisions taken around spreading COVID-19-related *awareness* in the general public (Figure 6(d)). We observe that overall the proportion of posts across the four countries discussing the COVID-19 awareness is somewhat comparable, with the United States leading the peer group with c.30% posts. However, there is a significant difference across countries in terms of expressed sentiments with the users from India leading the peer group with most positive outlook towards vaccination awareness (c.2x compared with Canada) followed by the United Kingdom and the United States. Next, we compare the four countries based on one of the most significant attributes related to COVID-19 vaccines, i.e. *hesitancy* (Figure 6(e)). We observe that while the users from all of the four countries were experiencing hesitancy issues to some extent, the situation was relatively most critical in the United States, which had a high proportion of posts discussing hesitancy issues and the most negative outlook compared with the rest of the three countries. On the contrary, the situation was relatively much stable in the United Kingdom, which had the lowest proportion of posts discussing hesitancy issues and the most positive outlook towards it compared with the rest of the countries. Finally, we compare the four countries on the basis of a key COVID-19 vaccine administration strategy, i.e. *prioritising* the vaccination of the individuals based on profession, health, and/or age group (Figure 6(f)). In this case, we observe that both India and the United States were relatively discontent with this strategy of prioritisation as implied by their relatively higher proportion of posts and lower sentiment scores. In contrast, Canada and the United Kingdom had relatively less proportion of posts discussing prioritisation and had higher overall sentiment scores, thereby implying that the users in those two countries were relatively more accommodating when it came to prioritising certain individuals over the others for COVID-19 vaccination.

**Figure 7.**
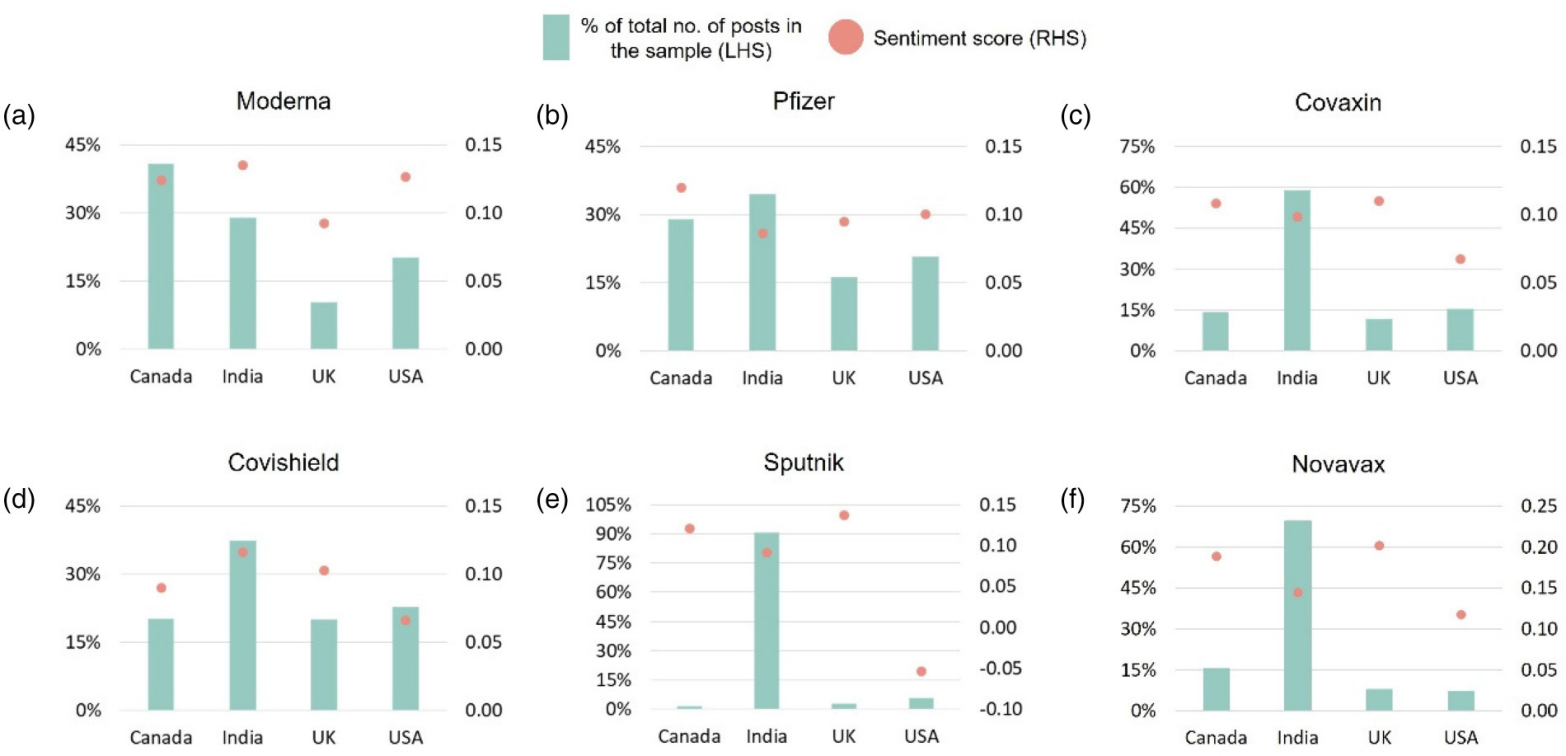
Comparing the sentiments towards the different COVID-19 vaccines across countries. On the left-hand side of the subplots in the *y*-axis, we show the percent of the total number of posts across countries in the respective samples collected from FAISS index using similarity search. And on the right-hand side, we show how the sentiment scores vary across countries on the basis of the different COVID-19 vaccines.

We also compare the sentiments towards the various available COVID-19 vaccines to assess the differences in their acceptance across the four countries (Figure 7). In the case of Canada, we observe that Moderna was the most discussed vaccine followed by Pfizer and Covishield, whereas the public sentiments towards all six vaccines largely stayed relatively positive. In the case of India, while all six vaccines had been discussed widely, the outlook on Moderna and Covishield was relatively more positive as compared with its peers. Notably, the posts related to Covaxin, Sputnik, and Novavax vaccines largely originated from India. When it comes to the United Kingdom, we observe that largely the posts pertain to Covishield followed by Pfizer, Covaxin, and Moderna in that order. However, when compared using sentiments, the United Kingdom had most positive outlook towards Covaxin followed by Covishield, Pfizer, and Moderna. Finally, comparing the sample from the United States, we observe that the posts mainly discussed four vaccines, with Moderna, Pfizer, and Covishield at somewhat similar level (20%) and Covaxin at a slightly lower proportion (15%). However, Moderna was the most favoured vaccine in the United States followed by Pfizer, whereas the public had relatively more concerns regarding Covishield and Covaxin.

### Models evaluation

**Figure 8.**
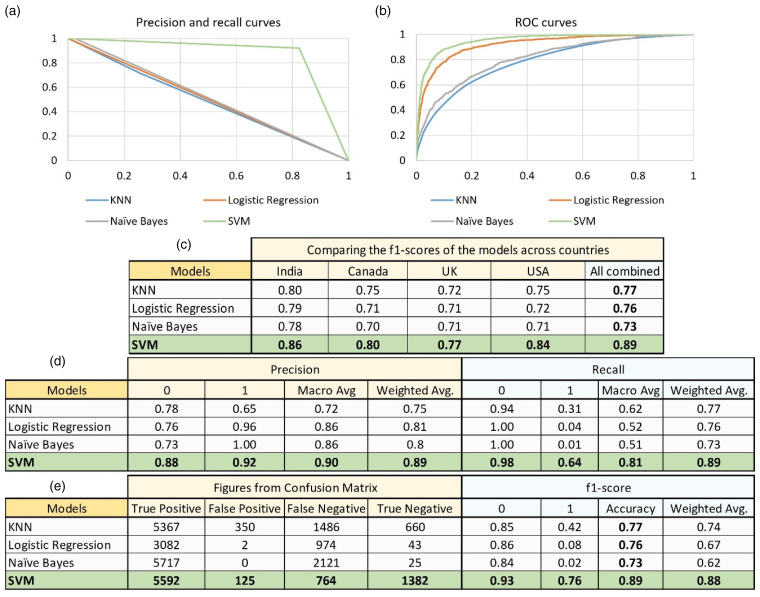
Performance comparison of the various machine learning binary classifiers. We provide the plots for precision and recall (a) and ROC (b) curves along with the output of the classification report and confusion matrix (d, e) for binary labels ‘0’ and ‘1’ for various supervised machine learning algorithms. Additionally, we validate our findings by comparing the accuracies of all the trained models across all four countries separately (c).

Finally, after splitting the labelled data into train-test sets (70:30), we train various supervised machine learning algorithms and validate their performance on the test set, and provide the relevant plots here (Figure 8(a) and (b)). SVM seems to perform the best on all the relative parameters among all the four algorithms tested (Figure 8(d) and (e)). With the five-fold cross-validation, it achieved the highest average accuracy of 89% and fared better compared with the other algorithms on both the labels ‘0’ and ‘1’ in terms of precision and recall. It was followed by KNN with an accuracy of 77%, but it had a very poor recall of only 0.31 for label ‘1’. Finally, the logistic regression achieved the accuracy of 76%, and the Naïve Bayes algorithms achieved the level of accuracy of 73% but had even poorer recall than that of KNN for label ‘1’ at 0.04 and 0.01, respectively.

In order to assess the validity of these machine learning algorithms, we train all four models on the country-level data separately and compare their performance based on f1-score (Figure 8(c)). In line with our previous findings, we observe that SVM consistently outperforms compared with the other models across all the four countries including India, Canada, the United Kingdom, and the United States, and combined. Hence, in terms of overall performance, SVM seems to be the best fit for the COVID-19 vaccines related data sets and may be suitable for production if the stakeholders are looking for a quick and ready-made solution.

## Discussions and conclusion

The sentiments and perspectives expressed by the Twitter and Reddit users in their posts on vaccines have been analysed with the use of machine learning framework. This study is one of the first to use topic modelling prediction classifiers to detect sentiment in posts connected to the COVID-19 vaccines with models validation done across four different countries viz. India, Canada, the United States, and the United Kingdom. It is vital to analyse and collate public views and opinions around COVID-19 vaccine discussions to prepare for more effective vaccination promotion in the future. The findings of this study revealed that optimism predominated over pessimism in conversations about the COVID-19 immunisation, and that trust and anticipation accounted for a significant portion of the emotions associated with fear. Word clouds, counts of word pairs, and correlations between words were used in the data visualisation for this research because of the unique insights that they bring into the results. For instance, common sentences and word combinations were presented in a manner that seemed very normal.

This study provides novel insights into the debates and perspectives of the four countries regarding COVID-19 immunisation. As a result of the expansion of the internet and social media, new routes for persuasion and the quick distribution of (false) information have been created. This presents possibilities as well as obstacles for the spread of vaccination information.^42^ To implement effective vaccination drives, public health authorities can monitor geo-aware and (near) real-time public opinion about vaccine-related material. Using the various analysis tools employed in this study, it is possible to monitor and implement effective regulation and promote participatory discourse to improve vaccination. Our results provide novel insights and policy suggestions for the purpose of preserving the social and economic health of a nation.

First, the results indicate that politicians have a substantial influence on the opinions of the general people against vaccination. Public figures who are against vaccination and who make negative comments about it could sway a substantial part of the community.^42^ People are more likely to believe the opinions of public figures because they are elected officials with the ability to modify healthcare systems and are perceived to have more knowledge about a vaccine.^39,40^ Therefore, public personalities have a duty to offer accurate information about. This highlights the need of doing research into the ways in which the involvement of notable persons in influencing the general public's perspective on vaccination.

Second, we show that the spread of anti-vaccination conspiracy theories resulted in a substantial drop in the overall sentiment ratings. We need to be wary of the fact that social media platforms with massive user bases have ‘disrupted’ conventional means of communicating vaccine information,^43^ making it easier for anti-vaccination campaigners to spread incorrect information. However, this also suggests that government authorities should investigate the possibility of utilizing these platforms to directly connect with residents regarding vaccination using geo-targeted messaging to address challenges that are specific to a certain place.

Third, assessments of both sensation and emotion revealed a wide variety of country-specific patterns. This allows us to identify those who have high negative sentiments and emotional anxieties and need more information relating to COVID-19 vaccination. In addition, we strongly recommend that governments and organisations working in the field of public health initiate COVID-19 immunisation campaigns in these areas to lessen the anxieties of the people living there and encourage them to be vaccinated. In line with the past studies, while distrust in the scientific community, side effects of the vaccines, and misinformation on the internet^25–29^ remain as some of the major challenges to lowering hesitancy, our study also shows that the immunisation campaigns should refrain from providing conflicting advice as it could fuel further hesitancy. Moreover, religious and cultural differences^85,86^ should be taken into consideration while planning such campaigns as different strategies may be better suited to the individuals from different cultures. Additionally, we find that factors such as old age, profession, level of education, overall health, and allergies can also influence the level of hesitancy of an individual towards vaccination and may be taken into consideration while designing different campaigns.

Fourth, we show that there is no clear winner between the countries with higher collectivism (strict cultures) vs. the countries with higher individualism (loose culture) when compared in the context of COVID-19. While one culture may have edge over the other in a specific situation – such as lower risk of diseases outbreak in the communities with loose cultures vs. higher rate of compliance in the communities with strict cultures^64^ – we observe that the advantages and disadvantages of the two cultures balance out each other in the long run.

Finally, we compare our results with that of the past studies to assess the similarities and differences among them. For instance, a study conducted on the similar tweets data set recently in 2023 argued that sentiments in favour of vaccination had increased over time.^87^ However, the underlying data set used in that study only had tweets until 2021. Hence, with this study, we extend the previous findings and show that positive sentiments towards vaccination were rising only during 2021 and experienced a gradual decline thereafter. Moreover, our findings related to COVID-19 vaccination hesitancy were mostly in line with the past studies^88,89^ with the majority of the concerns around side effects, mistrust, misinformation, culture, etc. However, we also show that conflicting advice, age, personal health, level of education, etc. can also play a crucial role in fuelling hesitancy. Furthermore, our findings related to the different classes of sentiments, i.e. positive, neutral, and negative, were consistent with the historical literature. Similar to a study conducted in 2021,^90^ our data sets had majority of the posts tagged as neutral (43.2%), followed by positive posts (41.2%) and a relatively lower count of negative posts (15.6%). Lastly, while we recommend the use of SVM algorithm to train the models on COVID-19 vaccines related data, a study conducted in 2023^87^ argued that extra tree classifier (ETC) using bag of words (BoW) outperformed the rest.

This study has a few limitations; considering that the majority of the users who make use of social media are young, the collected data set is not fully representative of the whole community and/or countries under consideration. Moreover, rather than being static, the geographical distribution of social media users is more often than not subject to shifts. It is necessary to take into account the problem of the ‘digital divide’, in addition to other technologically reliant research. This study examines only the responses of people who use Twitter and Reddit. As a result, it does not take into account the perspectives of certain demographics of the population, such as those living in rural areas (who may not have access to digital devices) or those who are reluctant to share their thoughts on social media platforms. In addition, by using the Twitter API, we were only able to obtain around 1% of all entries. Tweet disposition may be altered by going to different attractions during the course of the day, as shown by Padilla et al.^91^

In order to lessen the uncertainty and volatility of the sentiment scores and emotions brought on by the limitations discussed above, it will be necessary for future studies to raise the size of the sample. In addition to this, it is necessary to differentiate natives from visitors and conduct research at more localised and shorter time scales. Because emotion is a fluid and interrelated outcome of human experiences, it is possible that future research may focus on analysing additional, more diverse components of emotion in addition to the eight primary categories. In addition, the management of crises and disasters consists of four stages: the prevention stage (which involves building capacity), the readiness stage (which involves early warning), the response stage (which involves search, rescue, and emergency aid), and the recovery stage (which involves rehabilitation). The pandemic caused by the COVID-19 virus is still in the reactive phase of treatment. Researchers and medical professionals will be of great aid if they continuously monitor emotional and perspective shifts during the reaction and extend the length of the study to span the time of recovery or the main vaccination phase in the years following the pandemic.

In conclusion, the findings of the study provide grounds for optimism. Even though there are a lot of postings on vaccines on social media, most of the arguments in the mainstream media revolve around the question of whether vaccinations are safe, helpful in avoiding sickness, and lifesaving. It is a widespread misconception that immunisations protect against sickness. Our study highlights that since the internet and social media are so widespread today, corporate and government leaders are required to regularly engage the general people in risk awareness and engagement activities while also monitoring the dialogue and attitude that are prevalent on social media. According to the findings of this study, there has been a generally favourable shift in perspectives on the role of science and research in the field of immunisation. Yet, there is a rising lack of confidence as reflected in the negative and unfavourable posts. It is possible that fear is still the most prominent sensation, but when concerns about the COVID-19 vaccination rose, clusters of other bad feelings emerged. During a pandemic, it may be possible to speed up the process of finding widespread sentiment via the use of newly developed technologies as we demonstrated in our work.
